# Biocide-Resistant *Escherichia coli* ST540 Co-Harboring ESBL, *dfrA14* Confers QnrS-Dependent Plasmid-Mediated Quinolone Resistance

**DOI:** 10.3390/antibiotics11121724

**Published:** 2022-11-30

**Authors:** Srinivasan Vijaya Bharathi, Govindan Rajamohan

**Affiliations:** Bacterial Signaling and Drug Resistance Laboratory, Council of Scientific and Industrial Research—Institute of Microbial Technology, Sector 39-A, Chandigarh 160036, India

**Keywords:** commensal bacteria, reservoirs, drug resistome, active efflux, membrane transporters, outer membrane proteins

## Abstract

Emerging sequence types of pathogenic bacteria have a dual ability to acquire resistance islands/determinants, and remain renitent towards disinfection practices; therefore, they are considered “critical risk factors” that contribute significantly to the global problem of antimicrobial resistance. Multidrug-resistant *Escherichia coli* was isolated, its genome sequenced, and its susceptibilities characterized, in order to understand the genetic basis of its antimicrobial resistance.The draft genome sequencing of *E. coli* ECU32, was performed with Illumina NextSeq 500, and annotated using a RAST server. The antibiotic resistome, genomic island, insertion sequences, and prophages were analyzed using bioinformatics tools. Subsequently, analyses including antibiotic susceptibility testing, E-test, bacterial growth, survival, and efflux inhibition assays were performed.The draft genome of *E. coli* ECU32 was 4.7 Mb in size, the contigs were 107, and the G+C content was 50.8%. The genome comprised 4658 genes, 4543 CDS, 4384 coding genes, 115 RNA genes, 88 tRNAs, and 3 CRISPR arrays. The resistome characterization of ST540 *E. coli* ECU32 revealed the presence of ESBL, APH(6)-Id, APH(3′)-IIa, *dfrA14*, and QnrS1, with broad-spectrum multidrug and biocide resistance. Comparative genome sequence analysis revealed the presence of transporter and several virulence genes. Efflux activity and growth inhibition assays, which were performed with efflux substrates in the presence of inhibitor PAβN, exhibited significant reduced growth relative to its control.This study discusses the genotypic and phenotypic characterization of the biocide-tolerant multidrug-resistant *E. coli* O9:H30 strain, highlighting the contributory role of *qnrS*-dependent plasmid-mediated quinolone resistance, in addition to innate enzymatic modes of multidrug resistance mechanisms.

## 1. Introduction

Among the different members of genus *Escherichia*, serotypes from bacterial species *E. coli* that belong to *Enterobacteriaceae* are highly pathogenic for humans, birds, and animals. This Gram-negative bacterium is a rod-shaped, facultative anaerobe found in soil, food, environment, and the intestines of animals and humans, with an unmatched capacity to survive in diverse, stressful conditions. *E. coli* produces an arsenal of virulence factors, such as fimbrial adhesins, different iron acquisition systems, heat-labile/stable toxins and hemolysin, capsules, a type III secretion system, and colonization invasion factors. The toxin-producing *E. coli* strains are responsible for causing mild sicknesses, such as diarrhea and vomiting, to severe illnesses such as meningitis, respiratory diseases, pneumonia, and urinary tract infections [1,2]. As per the 2017–2018 GLASS report, *E. coli* was the most frequently reported pathogen with high-level resistance to ciprofloxacin and imipenem. Subsequently, in its 2019–2020 report, focusing on AMR data from 65 different countries, *E. coli* isolates tested from urine samples (from 64% countries) were resistant to ceftriaxone, cefotaxime, ampicillin, and ciprofloxacin, including carbapenems. In 2017, the WHO published its priority list of clinically significant human pathogens, and MDR *E. coli* was classified under the critical threat category, in order to ensure enhanced research activities to control AMR spread, and infection control [3,4]. 

The major pathotypes of *E. coli* include intestinal (IPEC) and extraintestinal (ExPEC) pathogenic strains. Of the ExPEC *E. coli*, UPEC (uropathogenic *E. coli*) evades the host’s innate immunity and colonizes the urinary bladder, kidneys, and is well recognized as a causative agent for urinary tract infections (UTIs), which account for ~80% of urinary infections [5,6]. 

Recently, India published its first pathogen priority list with a similar frame of objectives, carbapenem and tigecycline resistant *E. coli* topped the critical threat category. According to recent ICMR-AMR data, out of the total 107,387 isolates studied during the year 2019, the relative distribution of *E. coli* remained the highest at 28% (*n* =30822) in Indian hospitals from different locations. *E. coli* was the most predominant isolate from urine (56%), with strains exhibiting reduced susceptibility to cephalosporins and fluoroquinolones, including imipenem (from 86% in 2016 to 63% in 2019). While ST648, ST2659, and ST540 have been reported to be NDM-5-producing *E. coli* globally, the frequently reported ExPEC sequence types that are linked with UTI cases include ST131, ST73, ST95, and ST69 [7]. 

Primary susceptibility testing of *E. coli* urinary isolate ECU32 illustrated its antimicrobial resistance behavior, with an innate capability to co-produce multiple resistance determinants. The phylogenetic analysis classified this isolate to the ST540 serotype, one which is usually reported from birds and animals.Therefore, to reach a deeper perspective on its genetic content, genome organization, and phenotypic behaviors, asystematic study was initiated.This study reports the draft genome sequence, analysis, and resistome characterization of ST540 *E. coli* urinary isolate ECU32 from India, co-producing ESBL, APH(6)-Id, APH(3′)-IIa, *dfrA14*, and QnrS1, with broad multidrug and biocide resistance.

## 2. Results and Discussion

### 2.1. Genomic Features and Phylogenetic Analysis of E. coli 

The draft genome reads of *E. coli* ECU32 were assembled to a single chromosome of size 4.7 Mb, an N50 spanning 94, 946 bp, L50 being 15, number of contigs being 107, and a G+C content of 50.8% (Assembly: GCA_002872235.1; GenBank: LZGD01000000). Analysis revealed 4658 genes (total), 4543 CDS (total), 4384 coding genes, 115 RNA genes, 8, 3, 2, 5S, 16S, 23S rRNA genes, 88 tRNAs, and 159 pseudogenes (Table 1). Furthermore, PADLOC analysis revealed three CRISPR arrays, including cas_type_I-E array system (Table 2) [8].

**Figure 1:**
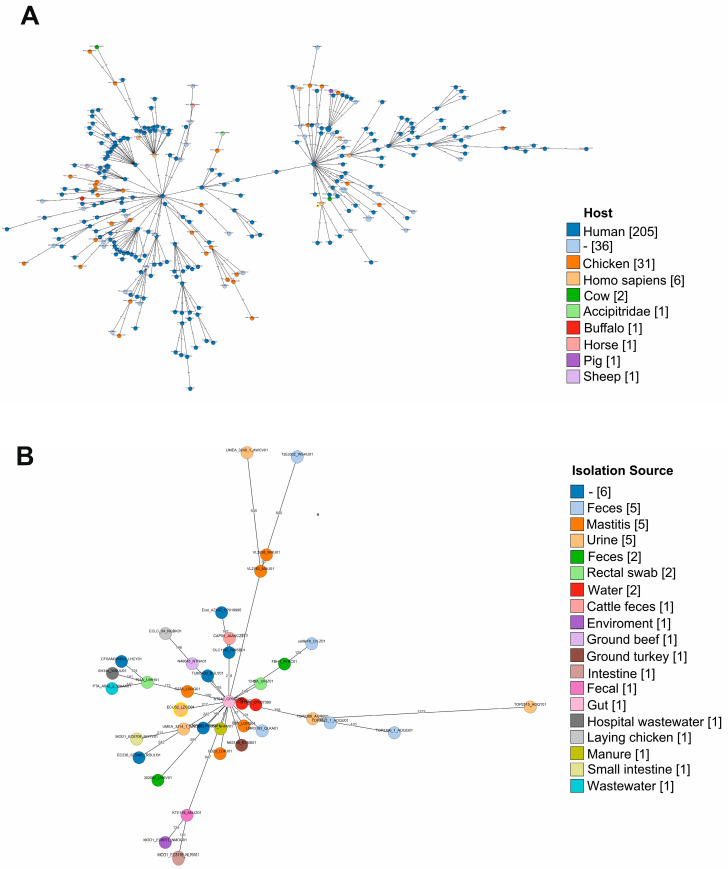

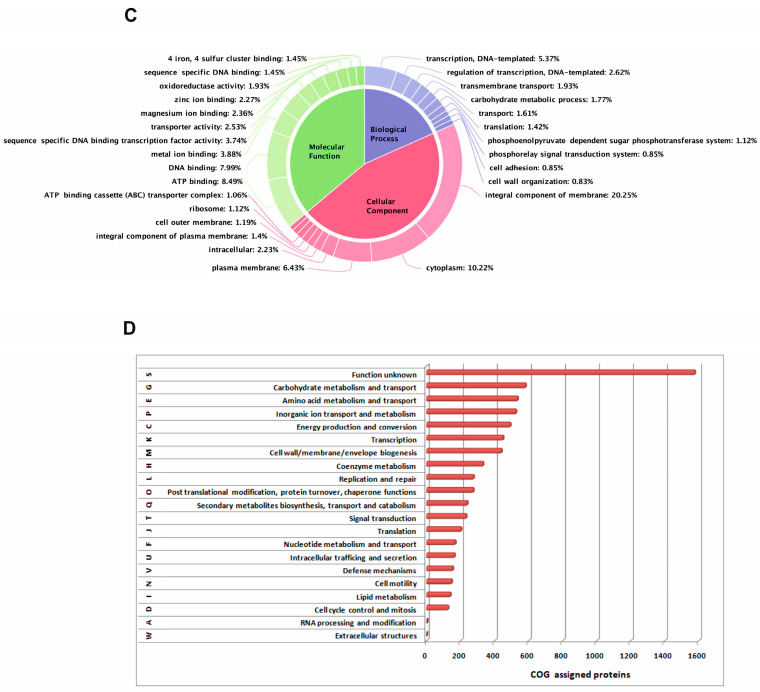
(**A**). The core genome multilocus sequence typing-based phylogenetic relationship tree was constructed for all of the available Indian *E. coli* strains in the BacWGSTdb server from different hosts, and ST groups were compared with the reference ST540 *E. coli*_ AZ147 strain. Grape tree displayed the relationship between isolates and comprised two major cluster groups. The *E. coli* ECU32 strain highlighted with a yellow circle with black center was found to be associated with human isolates in the second cluster. The lengths of all branches are scaled logarithmically. The numbers mentioned in square brackets are isolates from a range of hosts. (**B**). Grape tree represents the phylogenetic relationship of available *E. coli* ST540 strains in BacWGSTdb from different isolation sources and countries. The strain name was mentioned along the colored circle, the color indicates the source of isolation, and the isolate counts are mentioned in brackets. The *E. coli* ECU32 strain was highlighted with a yellow circle. The node label represents the allele differences, and the branch lengths are scaled logarithmically. (**C**). Gene ontology annotation of predicted genes was performed using BLAST analysis, and top gene ontologies were classified into cellular components, molecular functions, and biological process. (**D**). Distribution of clusters of orthologous groups (COG) in *E. coli* ECU32. The homologous gene clusters were functionally categorized and classified with COG assignments.

The genomic features of selected ST540 *E. coli* strains were investigated with Indian *E. coli* strains from different available sources in the BacWGSTdb server, while phylogenetic relationshipswere analyzed using the cgMLST approach with the ST540 reference strain. The analysis revealed two major clusters based on the isolation source; the ECU32 strain was found in the second cluster, along with strains predominantly isolated from humans (Figure 1A). Further analysis with only *E. coli* ST540 Indian isolate revealed its clustering with strains of human gut origin (Figure 1B). Overall, the cgMLST analysis revealed that the *E. coli* ECU32 strain was closely related to the human and gut origin multidrug isolates.

Additionally, in the RAST server, functional annotation of the genome revealed the presence of 595 subsystems (39% unassigned). The gene ontology data showed the distribution of genes for different components and functions. The biological processes includedgenes involved in transcription 5.37%, regulation of transcription 2.62%, transmembrane transport 1.93%, carbohydrate metabolic process 1.77%, cell adhesion 0.85%, cell wall organization 0.83%, and integral component of membrane 20.25%. The cellular components included cytoplasm with 10.22%, and the molecular function included genes with oxidoreductase activity 1.93%, zinc ion binding 2.27%, magnesium ion binding 2.36%, plasma membrane 6.43%, DNA binding 7.99%, ATP binding 8.49%, and transporter activity 2.53% (Figure 1C,D).

The clinical strain belonged to ST540 as the alleles *adk_6*, *fumC_7*, *gyrB_57*, *icd_1*, *mdh_8*, *purA_8*, and *recA_2* exhibited 100% identity to their respective locus, as per MLST typing. Based on Clermon typing, the strain belonged to phylogroup A, a group largely dominated with commensal origins of strains. The *E. coli* ECU32 belongs to serotype H30-H with 100% identity for *fliC* in scaffold8|size160413, *fimH54* (scaffold11|size140663), and O9-O antigen (>99% identity to *wzm* and *wzt* in scaffold34|size42957). These gene clusters were found between *hisI* and *gnd* genes, and belong to group 1 K antigens. 

### 2.2. Antimicrobial-Resistant Genes

Upon analyzing the sequence of *E. coli* strain ECU32 for genetic clusters, a 9852-base-pair genomic island was identified that carried the CRISPR-associated proteins, and the 5540-base-pair genomic island (scaffold94xsize3049) showed the presence of ESBL, the class-A *bla*TEM beta-lactamase (A8A11_02620). 

The 32,081-base-pair genomic island in the strain (scaffold7xsize174057) highlighted the presence of members from the EscJ/YscJ/HrcJ family type III secretion system (from A8A11_10065 to A8A11_10125). 

The largest genomic island present in strain ECU32, 131,243bp in size (consisting of scaffold78xsize4056 and scaffold83xsize2691), indicated the presence of aminoglycoside-modifying enzymes APH(3′) (A8A11_04060), APH(6)-Id (A8A11_04065),and class 1 integrase, followed by gene cassette *dfrA1* (A8A11_21925). The class-C *ampC* detected in this study exhibited 97% identity to a homolog found in *E. coli* O157:H7; 95% to *Shigella sonnei*; 78% to *Enterobacteriaceae* bacterium; 73% to *Shigella flexneri*; 71% to *Enterobacter*, and 70% to *Yersinia ruckeri*. 

The quinolone resistance gene *qnrS1* was found in scaffold57xsize16952 (A8A11_12295) with an adjacent ISKra4-like element (A8A11_12305), while the sulfonamide-resistant dihydropteroate synthase *sul2* was found in scaffold78xsize4056 (A8A11_04055). The quinolone resistance determinant exhibited identities to homologs found in *Vibrio mytili* CAIM 528 (95%); *Photobacterium ganghwense* strain DSM 22954 (92%); *Vibrionales* bacterium SWAT-3 (83%); and *Photobacterium halotolerans* strain MELD1 (66%). The sequence analysis of QnrS1 indicated the conserved pentapeptide sequences, and homology modeling highlighted the differences in loops A and B regions, as shown in Figure S1.

Homologs of well characterized efflux pumps, AcrAB, EmrAB, MdtABC, EmrE, AcrD, and TetA (scaffold82xsize4187; A8A11_05610), were present in the genome; moreover, the *marR* gene harbored sense mutations at the Y137H and G103S residues. The tetracycline efflux pump exhibited varied identities to homologs found in *Aeromonas simiae* CIP 107798 (98%), *Pseudomonas fluorescens* HK44 (97%), *E. coli* O83:H1 str. NRG 857C (97%), and *Clostridium nexile* DSM 1787 (78%).

**Figure 2:**
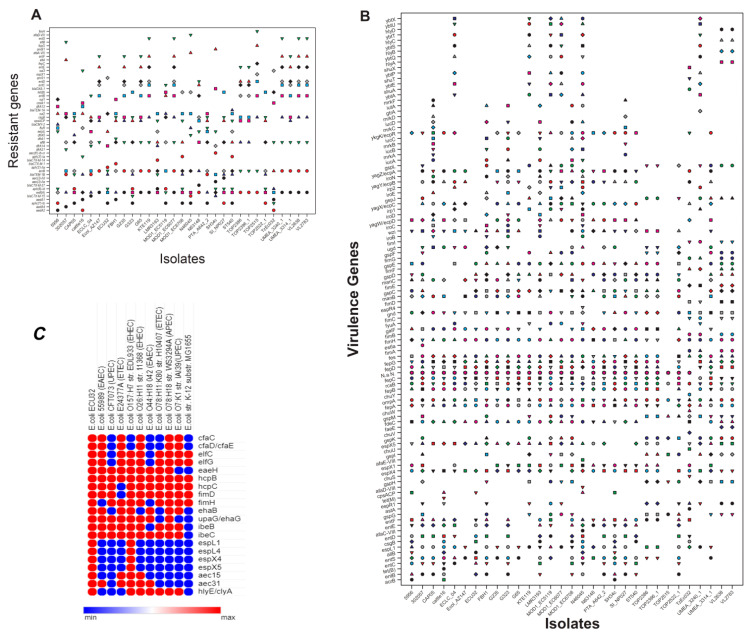
(**A**) Prevalence of resistance genes from different Indian *E. coli* ST540 strains, originating from various hosts. The strains used for analysis were strain 5956 (NHPV01, cow); strain 302057 (LRKV01, human); CAP05 (JAAKCZ01, cow); cattle16 (LVLZ01, cow); ECLC_04 (NQBK01, chicken); Ecol_AZ147 (CP018995, human); ECU32 (LZGD01, homo sapiens); FBH1 (AYRC01, human); G235 (LOOQ01, cow); G323 (LOPJ01, cow); G65 (LOPQ01, cow); KTE119 (ANUZ01, human); LMR3193 (QLKA01, human); MOD1_EC5119 (NLRV01, human); MOD1_EC6077 (NMDG01, Serpentes); MOD1_EC6708 (NOTV01, cow); N46045 (NTNA01); N63148 (NTMS01); PTA_A642_2 (WAAK01); SH34c (WSUU01); SI_NP027 (BGHG01, cow); ST540 (CP007265, human); TOP2386(AORB01, human); TOP2396_1 (AOQQ01, human); TOP2515 (AOQT01, human); TOP2522_1(AOQU01, human); TzEc032 (WSHU01, sheep); UMEA_3240_1 (AWCV01, human); UMEA_3314_1 (AWDE01, human); VL2638 (MIXJ01, cow); VL2783 (MIVJ01, cow). The strain accession numbers and hosts are mentioned in parenthesis. (**B**) The diversity of virulence factors are distributed in various Indian ST540 isolates. (**C**) The virulence factors of the ECU32 strain were compared with other *E.coli* pathotypes. The strains were *E. coli* 55989 (enteroaggregative *E. coli*—EAEC) [NC_011748]; *E. coli* CFT073 (uropathogenic *E. coli*—UPEC) [NC_004431]; *E. coli* E24377A (enterotoxigenic *E. coli*—ETEC) [NC_009801]; *E. coli* O157:H7 str. EDL933 (enterohemorrhagic *E. coli*—EHEC) [NC_002655]; *E. coli* O26:H11 str. 11368 (EHEC) [NC_013361]; *E. coli* O44:H18 042 (EAEC) [NC_017626]; *E. coli* O78:H11:K80 str. H10407 (ETEC) [NC_017633]; *E. coli* O78:H18 str. WS3294A (avian pathogenic *E. coli* APEC) [NC_020163]; *E. coli* O7:K1 str. IAI39 (UPEC) [NC_011750]; *E. coli* str. K-12 substr. MG1655 [NC_000913]. Min (blue) and Max (red) indicate absence and presence of virulence genes in a strain, respectively. Accession numbers are listed within the square brackets.

Using the BacWGSTdb server, the resistance genes were assessed amongst ST540 strains from different countries, hosts, and isolation sources, and the ECU32 strain exhibited the presence of diverse resistance genes (Figure 2A).

### 2.3. Virulence and Its Associated Genes

Comparative genome sequence analysis revealed that *E. coli* strain ECU32, in scaffold21xsize74584, carried ORFs for both the ferric enterobactin ABC transporter system (*fepA*, *fepB*, *fepC*, *fepD*, *fepG*, *fes*), and enterobactin (siderophore) biosynthesis exporter system (*entB*, *entC*, *entD*, *entE*, *entF, entS*). Important virulent factors that were identified included the type 1 fimbriae adhesin *fimH* (scaffold11xsize140663-1, A8A11_14300), hemolysin E *hlyE* (scaffold22xsize79984, A8A11_04330), invasin of brain endothelial cells, *ibeB* (scaffold60xsize15487, A8A11_08140), *gspC*, *gspD*, *gspE*, (scaffold35xsize41361), and intimin-like adhesion *fdeC* (scaffold1xsize239491 with 93% to EC958_0448).

Virulence genes were analyzed and compared with different ST540 strains and pathotypes. The analysis demonstrated the prevalence of virulence genes that are involved in adherence, autotransporter, invasion, non-LEE-encoded TTSS effectors, secretion system, toxin, and others (Figure 2B,C).

### 2.4. Phenotypic Characterization of Multidrug-Resistant E. coli

The Kirby Bauer assay revealed the *E. coli* strain ECU32 to be multidrug resistant (AMP, AMK, CLI, CST, ERY, KAN, LZD, MET, OXA, PEN, RIF, STR). The minimum inhibitory concentrations of this Indian isolate for different antibiotics, as evaluated by the agar dilution method, were ceftazidime 0.5 µg/mL, ticarcillin >1024 µg/mL, neomycin 4 µg/mL, norfloxacin 16 µg/mL, doxycycline 64 µg/mL, tetracycline 64 µg/mL, nalidixic acid >1024 µg/mL, ampicillin >1024 µg/mL, kanamycin 16 µg/mL, erythromycin >1024 µg/mL, trimethoprim 64 µg/mL, and chloramphenicol 4 µg/mL.

**Figure 3:**
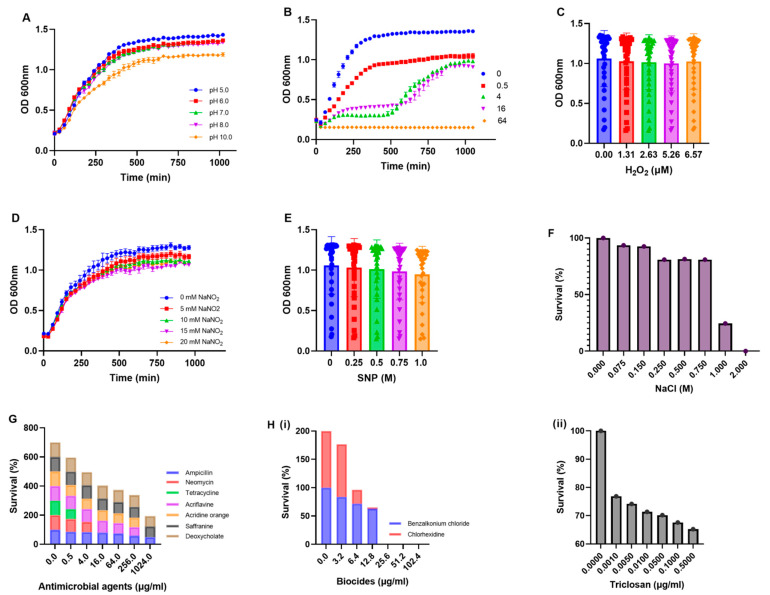
Biological characterization of the *E. coli* ECU32 strain. The growth profile of the *E. coli* ECU32 strain was monitored in the presence of different pHs (**A**) and concentrations of norfloxacin (**B**). The growth of the ECU32 strain was monitored in the presence of varied concentrations of oxidative stress-inducing agent—hydrogen peroxide (**C**) and nitrostative stress-inducing agents—sodium nitrite and sodium nitroprusside (**D**,**E**). The osmotic stress response survival was determined in the presence of sodium chloride concentrations (**F**). The survival ability of the *E. coli* ECU32 strain was determined under different concentrations of antibiotics [ampillicin, neomycin, tetracycline], bile [deoxycholate], structurally unrelated compounds and dyes [acriflavine, acridine orange, saffranine] (**G**), and biocides [benzalkonium chloride, chlorhexidine, and triclosan] (**H**(**i**,**ii**)). The mean values of the different independent experiments were used for plotting graphs, using GraphPad Prism.

*E. coli*, a common flora in the human gastrointestinal tract, has adaptive survival response strategies under different stress conditions. The growth profile of *E. coli* ECU32 was assessed in the presence of low to high pH concentrations. The strain retained the ability to grow in pH 5.0 to pH 8.0; meanwhile, in pH 10, the strain displayed less (~0.5-fold) growth compared to pH 5.0 (Figure 3A). Concentration-dependent growth of the strain in the presence of the antibiotic norfloxacin was observed. The strain was able to grow in doses upto 64 µg/mL, and exhibited nine-fold lower growth compared to its control (Figure 3B).

However, the *E. coli* ECU32 strain was able to survive in the presence of both oxidative and nitrostative stress-inducing agents, such as hydrogen peroxide, sodium nitroprusside, and sodium nitrite, respectively (Figure 3C–E). 

The survival of *E. coli* ECU32 under osmotic stress conditions was determined in the presence of different sodium chloride concentrations. The strain exhibited more than 80% survival upto 0.75 M, and 40% in 1 M of sodium chloride (Figure 3F). Altogether, these observations emphasize the adaptability of *E. coli* under any intracellular stress conditions, and its survival inside the host to cause disease severity.

The *E. coli* ECU32 strain has an ability to cope with different intracellular stress responses. Furthermore, the ability of the strains to survive in the presence of a various range of antimicrobial agents was examined. Analyses revealed a more than 50% survival rate was observed for the strain at 1024 µg/mL for ampicillin, 4 µg/mL for neomycin, and 0.5 µg/mL for tetracycline. Furthermore, the strain was found to have retained the ability to survive in various antimicrobial compounds, such as acriflavine, acridine orange at 256 µg/mL, and saffranine and deoxycholate at 1024 µg/mL (Figure 3G).

In order to detect efflux activity in the *E. coli* strain, MICs for the following efflux-based substrates were initially analyzed: acridine orange 256 µg/mL, acriflavine 64 µg/mL rhodamine >1024 µg/mL, saffranine >1024 µg/mL, deoxycholate >1024 µg/mL, and SDS >1024 µg/mL. Hence, a growth inhibition assay was performed with the *E. coli* strain ECU32, using ampicillin 256 µg/mL, in the presence of known efflux pump inhibitor PAβN, which exhibited an approximatelyfive-fold reduced growth relative to its control. Overall, the assays demonstrated that the strain has the ability to survive under different assails, including antimicrobial compounds. Furthermore, the sequence analysis and phenotypic efflux assays indicated the role of active efflux as the primary mechanism used to confer antimicrobial resistance. In order to substantiate these observations, genomic analysis of the strain further confirmed the presence of well-characterized efflux families, depicting the importance and possible involvement of these pumps in antimicrobial resistance and multiple cellular functions.

The strain was examined for its resistance level towards hospital-based disinfectants, and it exhibited tolerance as follows: benzalkonium chloride 12.8 µg/mL, chlorhexidine <3.2 µg/mL, and triclosan >0.1 µg/mL (Figure 3H(i,ii)), indicating that the urinary strain was broad-spectrum antimicrobial, as well as biocide-resistant. Additionally, the *E. coli* strain ECU32 displayed an ability to form biofilms (ratio 570/600 nm = 0.252), as it harbored genes that are required for adhesion and virulence.

Upon transforming the plasmid preparation from the MDR strain into *E. coli* JM109, transformants were confirmed on ampicillin (>256 µg/mL) and ciprofloxacin (0.5 µg/mL) plates; subsequent PCR detection indicated the presence of quinolone resistance determinant in the transformant, and further sequencing confirmed the role of qnrS-dependent plasmid-mediated resistance in *E. coli* strain ECU32. 

It is worthwhile to state here that ST540 has usually been reported in *E. coli* that was isolated from birds and chickens. Identifying the serotype ST540 from a biological sample strictly emphasizes the periodic monitoring of emerging *E. coli* strains at the One-Health Interface. The *E. coli* strains TOP2386 (accession no: AORB01), TOP2396_1 (accession no: AOQQ01), TOP2515 (accession no: AOQT01), TOP2522_1 (accession no: AOQU01), and TUM3433 (accession no: BGLY01), isolated from human samples in USA and Japan, belong to the same ST, as that of *E. coli* strain ECU32. As per the BacWGSTdb server, *E. coli* strain ECU32 (accession no: LZGD01) with ST540 is being reported for the first time in India. 

## 3. Materials and Methods

### 3.1. The Genome Draft Sequence, Annotation and Analysis 

The *E. coli* strain, ECU32, selected for this analysis, was obtained in 2013 from a urine sample during our longitudinal study. Draft genome sequencing was carried out with paired-end sequencing technology, Illumina NextSeq 500, and scaffold annotation was performed using a RAST server [9]. Basic local alignment search tool, (BLAST) (GO), was utilized for determining percent identities [10]. The *E. coli* ECU32 genomesequence was compared with available *E. coli* genomes (*n* =300) that were isolated from India, and core genome multilocus sequence typing (cgMLST) was performed with the selected reference genome (*E. coli*_ AZ147 GenBank CP018995 ST540) for phylogenetic analysis. The graph tree and minimum spanning tree were constructed and analyzed, based on cgMLST profiles of Indian *E. coli* isolates. The antibiotic resistome, genomic island, insertion sequences, and prophages were analyzed using bioinformatic tools such as Pathosystems Resource Integration Center tool, Resistance Gene Identifier, Island Viewer 4, Mobile Element Finder, PHAge Search Tool Enhanced Release, and NCBI-BLAST [9,10,11]. The CRISPR was determined using PADLOC server (https://padloc.otago.ac.nz/ accessed on 26 September 2022). Comprehensive Antibiotic Resistance Database (CARD; https://card.mcmaster.ca/analyze/rgi accessed on 10 May 2021) was used to predict AMR genes and the VR2 profile, in order to identify the associated mobile elements [8,12,13]. Virulence, transposons, and antibiotic resistance genes were determined using the BacWGSTdb server. The distribution of virulence factors in *E. coli* ECU32 with other *E.coli* strains was deciphered using virulence factor database analyzer [14].

### 3.2. Antibiotic Resistance Pattern and Growth Analysis

The antibiotic susceptibility testing, E-test, survival assays, stress response, growth curves, and efflux inhibition assays were performed, as described previously [9,11]. 

### 3.3. Data Deposition 

The *E. coli* ECU32 whole genome sequence was submitted to GenBank NCBI, with accession number LZGD00000000.1.

## 4. Conclusions

Overall, this study highlighted the presence of diverse virulence factors and the antibiotic resistome in ST540 *E. coli* strain ECU32, emphasizing a pressing need to conduct large-scale epidemiological surveillance to monitor the dominant ST/strain that is circulating within the cohort, and also to track the changing patterns in antibiotic susceptibility and resistome amongst swiftly disseminating bacteria such as *E. coli* in India. Emerging STs remain renitent towards hospital sterilization protocols, and havean unprecedented ability to acquire additional resistance determinants; therefore, they are “critical risk factors” that contribute significantly to the global problem of antimicrobial resistance. 

## Figures and Tables

**Table 1:** Genomic features of *E. coli* ECU32.

Type	Assembly Statistics
Genome	*Escherichia coli* ECU32
Size	4,734,193
GC content	50.8
Number of coding sequences	4601
Number of RNAs	105
Number of subsystems	595
Contigs generated	107
Maximum contig length	239,491
Minimum contig length	502
Average contig length	44,244.8 ± 55,998.5
Median contig length	8912
Total contigs length	4,734,193
Total number of non-ATGC characters	663
Percentage of non-ATGC characters	0.014
Contigs ≥ 500 bp	107
Contigs ≥ 1 kbp	97
Contigs ≥ 10 kbp	67
Contigs ≥ 1 Mbp	0
N50 value	95,844
L50	15
Genome coverage	93.0X

**Table 2:** Probable CRISPR systems in *E.coli* ECU32 using PADLOC analysis.

CRISPR System	Protein	Target	Sequence Id	Start	End	Strand
CRISPR_array	CRISPR_array	CRISPR001	LZGD01000013.1	92,385	92,901	-
cas_type_I-E	Cas2e	A8A11_20845	LZGD01000013.1	93,006	93,291	-
cas_type_I-E	Cas1e	A8A11_20850	LZGD01000013.1	93,292	94,210	-
cas_type_I-E	Cas6e	A8A11_20855	LZGD01000013.1	94,225	94,825	-
cas_type_I-E	Cas5e	A8A11_20860	LZGD01000013.1	94,811	95,486	-
cas_type_I-E	Cas7e	A8A11_20865	LZGD01000013.1	95,488	96,580	-
cas_type_I-E	Cas11e	A8A11_20870	LZGD01000013.1	96,592	97,075	-
cas_type_I-E	Cas8e	A8A11_20875	LZGD01000013.1	97,067	98,576	-
CRISPR_array	CRISPR_array	CRISPR002	LZGD01000043.1	123,708	123,910	-
retron_I-C	RT-Toprim_I-C	A8A11_16640	LZGD01000043.1	170,971	172,729	-
retron_I-C	msr-msd	NA	LZGD01000043.1	172,765	172,895	-
RM_type_II	MTase_II	A8A11_21460	LZGD01000051.1	19,587	21,006	+
RM_type_II	REase_II	A8A11_21465	LZGD01000051.1	20,986	21,457	+
DMS_other	BrxD	A8A11_09690	LZGD01000074.1	2659	3976	+
DMS_other	BrxHI	A8A11_09695	LZGD01000074.1	3972	6168	+
CRISPR_array	CRISPR_array	CRISPR003	LZGD01000087.1	154,485	155,734	+

## Data Availability

The genome sequence data have been deposited in NCBI GenBank accession number LZGD01000001 - LZGD01000107.

## Supplementary Materials

The following supporting information can be downloaded at: https://www.mdpi.com/article/10.3390/antibiotics11121724/s1, Figure S1: Sequence and homology modelling of QnrS.
