# Supplementary material for: Biocide-Resistant Escherichia coli ST540 Co-Harboring ESBL, dfrA14 Confers QnrS-Dependent Plasmid-Mediated Quinolone Resistance
Source: Antibiotics (Basel). 2022 Nov 30;11(12):1724. doi: 10.3390/antibiotics11121724 (PMC9774513; doi:10.3390/antibiotics11121724)
Supplement: Supplementary file 1 [file antibiotics-11-01724-s001.zip › antibiotics-1974885-supplementary.pdf]

A

METYNHTYRH **HNFSH KDLSD LTFTA CTFIR SDFRR ANLRD**

TTFVN CKFIE QGDIE G CHFDV ADLRD **ASFQQ CQLAM ANFSN**

ANCYG IEFRA CDLKG ANFSR TNFAH QVSNR MYFCS **AFISG**

CNLSY ANMER VCLEK CELFE NRWIG TNLAG ASLKE SDLSR

GVFSE DVWGQ FS **LQGAN LCHAE LDG** LDPRKVDTSGIKIAAWQ

QELILEALGIVVYPD

B

C

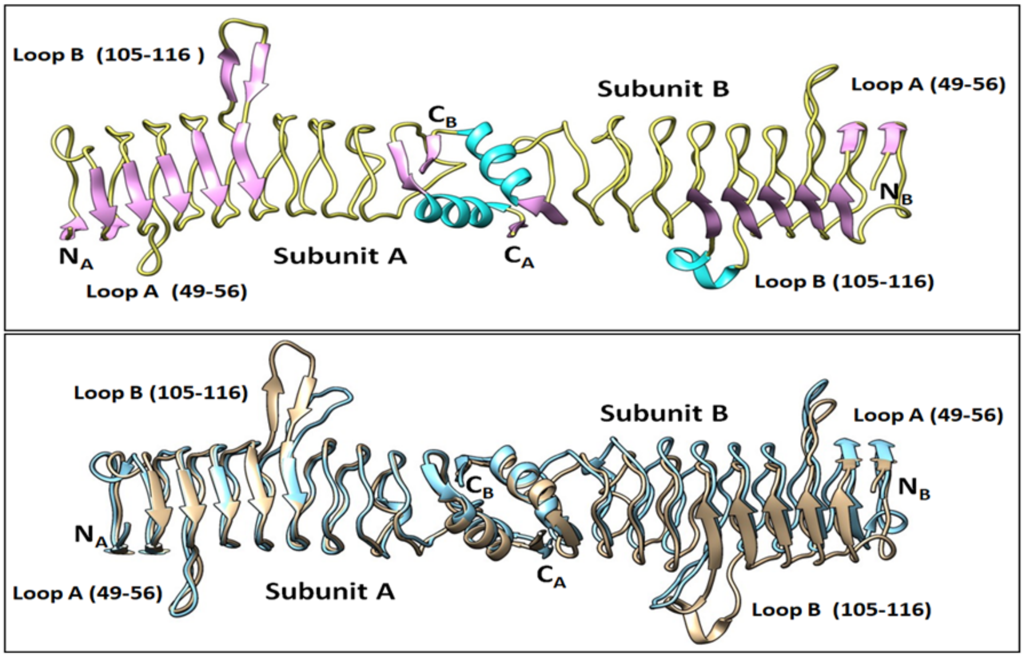

Figure S1. Sequence and homology modelling of QnrS.

A. Amino acid sequence of ECU32 QnrS1 display the consensus sequence of S/T/A/V/C-D/N-L/F-S/T/R-G, pentapeptide repeating unit residues shown as bold face and the underlined residues indicate loop regions of A and B.

B. Homology model of homodimer ECU32 QnrS1 using template of QnrB1 (PDB: 2xtx.1)

C. Superimposition of modeled homodimer ECU32 QnrS1 (light orange color) with AhQnr (light blue color) , the Qnr protein from *Aeromonas hydrophila* (PDB:3psz.1).
